# The inverted gender pyramid in career progression among GCC physicians: implications for health workforce policy

**DOI:** 10.3389/fmed.2026.1816552

**Published:** 2026-05-22

**Authors:** Salman Alzayani, Khaldoon Al-Roomi, Amer Almarabheh, Ali M. Hamdi

**Affiliations:** 1 Department of Family and Community Medicine, College of Medicine and Health Sciences, Arabian Gulf University, Manama, Kingdom of Bahrain; 2 Deanship of Students Affairs, Arabian Gulf University, Manama, Kingdom of Bahrain

**Keywords:** career progression, GCC physicians, gender equity, health workforce, leadership pipeline, medical graduates, promotion disparities, workforce policy

## Abstract

**Introduction:**

Gender equity in the health workforce is central to health systems policy and planning. In the Gulf Cooperation Council (GCC) countries, women constitute most of medical graduates, yet their representation at advanced medical career levels is lower than men, reflecting an inverted gender pyramid. Quantitative evidence across GCC countries remains limited. We examined the gender differences in physicians’ career progression among medical graduates of a regional medical school serving multiple GCC countries.

**Methods:**

A cross-sectional observational analysis was conducted using a pre-existing dataset of medical graduates from the College of Medicine and Health Sciences at Arabian Gulf University, Bahrain. Eligible records required gender, nationality, career level, and years since graduation. The sample included 685 graduates. Career progression was defined as advanced versus entry or mid-career level. Characteristics were compared by gender in bivariate analysis using chi square tests. In addition, adjusted odds ratios were estimated using multivariable logistic regression. A *p* < 0.05 was considered statistically significant.

**Results:**

Females comprised most of the medical graduates (57.7%). However, male physicians more frequently achieved advanced career levels than their female counterparts (50.3% vs. 43.0%, *p* = 0.027), with no significant gender differences in career years (*p* = 0.518). In adjusted logistic regression model, male physicians had higher odds of attaining advanced career progression compared with females (OR 2.253, 95% CI 1.377–3.686, *p* = 0.001).

**Discussion:**

An inverted gender pyramid was apparent, with women overrepresented among medical graduates but underrepresented among advanced medical career levels. The pattern is consistent with growing international evidence of gender inequities in the medical profession. There is an urgent need for health policy action that improves promotion transparency and formalizes leadership pipeline progression.

## 1 Introduction

Gender equity in the health workforce is increasingly recognized as a core health systems priority, with implications for workforce planning, leadership representation, institutional governance, and the equitable delivery of care. Globally, while women constitute the majority of medical graduate as well as the health and social care workforce, they remain underrepresented in senior healthcare positions (1, 2). This leadership gender gap is a health policy issue rather than an organizational matter because leadership composition influences strategic priorities, workforce retention, workplace culture, and the fairness of equal opportunity (1, 2). Evidence suggests that women’s leadership can be associated with measurable organizational benefits, including strengthened team processes and more inclusive decision making. Thus, women underrepresentation is not only inequitable but also inefficient for health systems (3).

In medicine, the last two decades have seen a sustained rise in women’s participation across different geographical regions. This process of feminization of the medical profession is due to the expanded access to medical education and changing societal expectations regarding women’s professional roles (4). However, the proportion of women seems to decline along the academic and managerial hierarchy, with fewer women at senior clinical leadership, department chair, and executive levels. A systematic review focusing on women’s leadership in academic medicine concluded that women remain underrepresented across leadership positions (5). This pattern indicates that the mere increase in the number of women entering the profession does not necessarily lead into a higher female representation in senior medical roles.

Understanding gender differentials in physicians’ career advancement is essential for evidence informed health workforce planning, leadership pipeline development, and equity-oriented retention strategies in Gulf Cooperation Council (GCC) healthcare systems. In these countries, the health sector has expanded rapidly alongside major investments in medical education, specialist medical training, and public sector health services. Consequently, women’s participation in medicine has increased substantially, and several country reports show that women constitute a larger share of medical students and entry level career physicians. For example, Oman has reported a rising number of female physicians in the health sector workforce (6). In Bahrain, the gender distribution across medical schools and health institutions documented a strong contribution of women to the physician workforce while leadership representation remained uneven, indicating that career progression may not be proportional to entry into the profession (7). These country specific observations align with an inverted gender pyramid. In this pattern, women are well represented among medical graduates and entry level career cohorts, but their representation diminishes progressively at advanced career levels, while men occupy a significantly greater share of senior roles. Qualitative studies from Saudi Arabia have highlighted that women who reached senior health leadership positions often navigate multilevel constraints, including organizational norms, limited transparency in leadership appointment processes and persistent gendered expectations regarding work and family roles (8, 9). Similarly, it appears that women trainee physicians in Saudi Arabia face workplace challenges during early career stages, particularly stress, discrimination, and harassment, which can influence career trajectories and willingness to pursue leadership pathways (10, 11).

Several knowledge gaps remain within the GCC context. First, much of the available regional evidence is qualitative or specialty specific, which limits the ability to generalize patterns across GCC countries and career stages. Second, while published research often emphasizes experiences of gender discrimination or barriers, studies do not show whether these experiences are translated into differences in career level achievements.

The present study addresses these knowledge gaps by analyzing a GCC physician dataset that includes gender, nationality, career level, and experience measures. The analysis focuses on whether an inverted gender pyramid is present in the post-graduation career progression and whether gender disparities persist after accounting for plausible confounders.

The research question addressed in this study: In GCC countries, is physician’s gender associated with career progression, as reflected by their achievements of advanced career levels?

This study aims to examine gender disparities in career progression among GCC physicians and to quantify whether an inverted gender pyramid exists from attaining the medical degree to advanced career attainment.

## 2 Materials and methods

### 2.1 Study design

This study employed a cross-sectional observational design using a preexisting, institution linked dataset of GCC medical students who graduated from College of Medicine and Health Sciences (CMHS) at Arabian Gulf University (AGU) in Bahrain.

### 2.2 Inclusion and exclusion criteria

Inclusion criteria were: (i) records belonging to CMHS-AGU graduates with Doctor of Medicine (M.D.) degree, (ii) availability of core demographic and workforce fields required for analysis (gender, nationality, and career level), and (iii) a valid value for years since graduation and year of graduation when complete case analyses were conducted. Exclusion criteria were: (i) duplicate records, (ii) records with missing values. Following this approach, the complete case analytic sample comprised of 685 subjects who graduated with M.D. degree.

### 2.3 Data collection

The dataset represented a structured compilation of physician workforce information available through AGU alumni records and follow up updates maintained in the study database. Data were extracted into an analysis ready spreadsheet and checked for internal consistency prior to statistical testing. Verification of permissible category values for each variable was undertaken by reviewing frequency distributions to detect any out of range values and ensuring consistent labeling of nationalities and career levels.

### 2.4 Study variables

Variables were gender (female, male), nationality (Bahrain, Saudi Arabia, Kuwait, Oman, and other), career level attainment (entry, mid, and advanced levels), and years of experience in the medical career. Years of practice in the medical profession were categorized into four groups (0–5, 6–10, 11–15, and > 15 years) to facilitate interpretable workforce descriptions and comparisons across career stages. Career level was categorized into three groups: entry, mid, and advanced levels. Entry level referred to physicians in early professional roles, including interns, junior residents or trainees. Mid-level referred to physicians who had completed postgraduate training or were practicing in intermediate professional roles, including specialists, senior residents or registrars. Advanced level referred to physicians occupying senior clinical, academic, administrative, or leadership roles, including consultants, department heads or program directors.

### 2.5 Statistical analysis

Statistical analyses were conducted in three stages levels: univariate, bivariate, and multivariable. Univariate analysis summarized study variables using frequencies and percentages. Bivariate associations between categorical variables were tested using chi square test. Core tests included gender by career level, gender by nationality, and nationality by career level. A *p* < 0.05 was considered statistically significant.

For multivariable analysis, career level progression (entry/mid, advanced) was modeled using logistic regression (reference category: entry/mid-level), with covariates including gender, nationality, and years in career. Results were expressed as adjusted odds ratios (OR) with 95% confidence intervals (CI) and *p*-values. If the CI did not include the value of 1.0, it was considered statistically significant. Data were analyzed using SPSS (version 30.0).

### 2.6 Ethical considerations

This study used a preexisting dataset of physician workforce information. Analyses were performed without identification of medical graduates’ names. The study involved no direct participant contact and no intervention. The study was approved by the Research and Ethics Committees in the CMHS at AGU (reference number: E28-PI-02-26).

## 3 Results

Table 1 presents the demographic characteristics of the sample. Most of the medical graduates were females (57.7%). The nationality distribution of the doctors was as follows: Bahrain (39.6%), Saudi Arabia (37.2%), Kuwait (13.9%), Oman (3.9%), and other nationalities (5.4%). Career level showed that 46.1% of the graduates have progressed into advanced career levels.

**TABLE 1:** Demographic characteristics of the medical graduates (*N* = 685).

Characteristics	Number	%
Gender		
Male	290	42.3
Female	395	57.7
Nationality		
Bahrain	271	39.6
Saudi Arabia	255	37.2
Kuwait	95	13.9
Oman	27	3.9
Other	37	5.4
Career level		
Entry	314	45.8
Mid	55	8.0
Advanced	316	46.1
Career years		
≤5 years	81	11.8
6–10 years	264	38.5
11–15 years	116	16.9
>15 years	224	32.7

**FIGURE 1:**
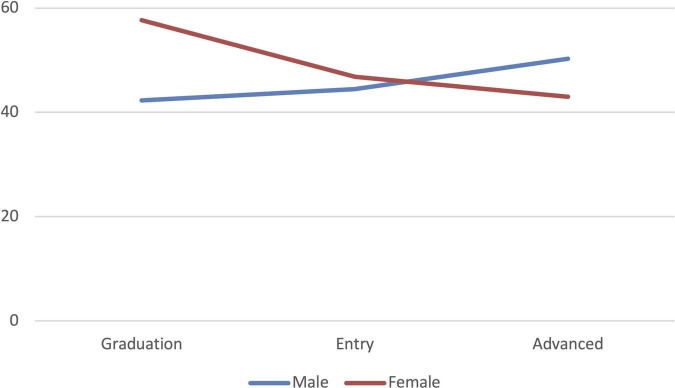
Gender distribution of doctors across medical career levels (*N* = 685).

Table 2 presents chi square comparisons between males and females by nationality, career level, and career years. Country distribution differed significantly by gender (χ^2^ = 18.982, *p* < 0.001), with a higher proportion of females from Bahrain (42.5% vs. 35.5%) and a higher proportion of males from Saudi Arabia (44.8% vs. 31.6%). It is interesting to find a statistically significant gender ratio inversion during the medical career progression with male doctors more frequently achieving advanced career levels compared to females (50.3% vs. 43.0%), *p* = 0.027. This gender disparity is not due to differences in career years (χ^2^ = 2.273, *p* = 0.518). The line graph (Figure 1) demonstrates the shift in gender pattern across the medical career, with female doctors graduating in greater numbers but males becoming more dominant at advanced career levels.

**TABLE 2:** Comparisons between the demographic characteristics of male and female doctors (*N* = 685).

Characteristics	Gender		χ^2^-value	*P*-value
	Male (*n* = 290)	Female (*n* = 395)		
Nationality				
Bahrain	103 (35.5)	168 (42.5)	18.982	< 0.001*
Saudi Arabia	130 (44.8)	125 (31.6)		
Kuwait	33 (11.4)	62 (15.7)		
Oman	15 (5.2)	12 (3.0)		
Other	9 (3.1)	28 (7.1)		
Career level				
Entry	129 (44.5)	185 (46.8)	7.249	0.027*
Mid	15 (5.2)	40 (10.1)		
Advanced	146 (50.3)	170 (43.0)		
Career years				
≤5 years	40 (13.8)	41 (10.4)	2.273	0.518
6–10 years	107 (36.9)	157 (39.7)		
11–15 years	51 (17.6)	65 (16.5)		
>15 years	92 (31.7)	132 (33.4)		

*Statistically significant.

Table 3 reports adjusted odds ratios from a multivariable logistic regression model including country, career level, and career years. Male doctors were significantly more likely to progress into advance career levels compared to their female counterparts (OR = 2.207; 95% CI = 1.346**–**3.619; *p* = 0.002). When using ≤ 5 years as the reference category, it was found that doctors who completed 11**–**15 years in their medical career had OR = 0.445; 95% CI 0.231**–**0.856; *p* = 0.015. The comparative figure for those doctors in the other career years categories were as follows: > 15 years (OR 0.400; 95% CI 0.202**–**0.792; *p* = 0.009) were associated with lower odds, while 6**–**10 years was borderline (OR 0.606; *p* = 0.056).

**TABLE 3:** Multivariable analysis of the relationships between career characteristics and gender (*N* = 685).

Characteristics	Category	Adjusted odds ratios	95% Confidence intervals (CI) (lower, upper)	*P*-value
Nationality	Bahrain	1.0		
	Saudi Arabia	1.815	1.254, 2.627	0.002*
	Kuwait	0.889	0.541, 1.460	0.641
	Oman	2.106	0.935, 4.744	0.072
	Other	0.499	0.223, 1.117	0.091
Career level	Entry/mid	1.00		
	Advanced	2.207	1.346, 3.619	0.002*
Career years	≤5 years	1.00		
	6–10 years	0.606	0.362, 1.014	0.056
	11–15 years	0.445	0.231, 0.856	0.015*
	>15 years	0.400	0.202, 0.792	0.009*

*Statistically significant.

Table 4 presents a logistic regression model that includes gender, nationality, and career years as independent variable while career progression into advanced or entry/mid-levels are the dependent variables. The analysis reconfirmed the finding that doctors who achieved advanced career levels were significantly more likely to be males (OR 2.253; 95% CI 1.377**–**3.686; *p* = 0.001) rather than females.

**TABLE 4:** Multivariable analysis of the relationships between the demographic characteristics and career level (*N* = 685).

Characteristics	Categorical	Adjusted odds ratios	95% Confidence intervals (CI) (lower, upper)	*P*-value
Gender	Female	1.0	1.377, 3.686	0.001*
	Male	2.253		
Nationality	Bahrain	1.0	0.560, 1.499	0.726
	Other	0.916		
Career years	≤5 years	1.00	0.464, 2.505	0.889
	6–10 years	1.079		
	11–15 years	17.094	7.384, 39.575	<0.001*
	>15 years	165.570	65.026, 421.576	<0.001*

*Statistically significant.

## 4 Discussion

This study provides clear quantitative evidence consistent with an inverted gender pyramid in career progression among physicians in the GCC countries. Women doctors constituted the majority overall of medical graduates and entry career level, yet men were more frequently represented in advanced career levels. The bivariate analysis showed a statistically significant association between gender and attaining higher career levels. In the multivariable analysis, even after accounting for years of experience and nationality, male gender remained associated with advanced career attainment. This observed disparity is not explained by differences in years since graduation alone. Taken together, these important findings add to the growing evidence of gender inequalities in healthcare workforce and demonstrate measurable differences in advancement to leadership positions.

Plausible explanations for these gender disparities in post medical graduation advancement in the GCC countries are most likely to be multi factorial and reflect a combination of structural, organizational, and social realities. First, differential access to high visibility assignments can influence promotion opportunities. Evidence from academic medicine consistently identify sponsorship as a particularly important factor to access leadership roles, nominations, and accelerated advancement (12). Second, gendered perceptions regarding caregiving and household responsibilities can shape employment patterns, specialty selection, and willingness or ability to pursue senior roles. Published reports emphasizes that women frequently suffer from the dilemmas of choosing priorities between professional advancement and social expectations, particularly the timing of childbearing and unequal distribution of domestic labor, which would negatively impact their promotion readiness (13). In GCC contexts, these dynamics are further amplified by cultural norms and institutional practices that do not systematically support flexible pathways for childcare provision, or equitable parental leave. Third, health workplace and exposure to discrimination or harassment may have also contributed to this gender disadvantage. In Saudi residency settings, studies have documented a high prevalence of harassment and discrimination among residents and were associated with negative effects on wellbeing and performance (14). It would appear that hostile training environments can influence career aspirations and reduce self-efficacy. Fourth, work segregation within medical specialties can shape who becomes eligible for senior positions. If women are underrepresented in specialties with greater leadership posts, an inverted gender pyramid will emerge as a subsequent outcome (15).

Comparison with other evidence from the GCC and the rest of the world supports the plausibility of these mechanisms. In Kuwait, a case study of leadership positions in government hospitals found substantial transitions over time in women’s representation in leadership roles, with some hospitals reaching even majority female leadership in specific periods (16). This shows that gender gap in the medical workforce may change with roper policy implementation along with institutional reforms. In parallel, regional qualitative evidence from the Middle Eastern countries indicates that women’s advancement remains constrained by cultural norms and organizational practices (17). The present study’s finding that gender disparities persist after accounting for career years aligns with the view that barriers operate throughout the career course and that the number of years in medical practice alone are insufficient to achieve equity in senior position representation.

Internationally, our results are consistent with increasing evidence that women remain underrepresented in leadership across academic and clinical hierarchies. A survey in the United States of America academic medicine found persistent gender differences in rank and leadership even after accounting for relevant factors, indicating that structural and institutional factors lead to unequal outcomes (15). Inequities persist across a wide range of leadership positions and that organizational level changes are required to address them (18). In addition, attrition from academic medicine is higher among females, which has progressively reduced the pool of women eligible for senior appointments, thereby reinforcing the inverted gender pyramid (19).

This study has policy implications which follow the observed inverted gender pyramid in GCC medical workforce. First, promotion governance must be made more transparent and auditable. Routine reporting of gender indicators, such as time to first supervisory role, time to senior appointment, and representation in key committees, can transforms equity goals into measurable accountability. Second, leadership pipeline programs should be structured and evaluated since mentorship alone is insufficient (20). Third, healthcare systems should address workplace climate and psychological safety. The presence of documented harassment and discrimination in residency programs in the GCC region indicates a need for an efficient reporting system (20).

Fourth, it is acknowledged that national workforce policies which affect hiring, promotion, and leadership appointments may create different opportunity structures for women and men. Policy reviews across GCC countries could identify governance arrangements that support equitable advancement. Fifth, data systems should be enhanced and strengthened. Workforce planning and policy require longitudinal, routinely updated datasets that allow tracking of doctors cohorts from medical graduation through senior roles.

Published GCC literature shows that structural barriers, rather than individual preference alone, contribute to slower progression for women in career tracks. A study on women in oncology in GCC countries found that female doctors face unequal access to leadership opportunities, limited visibility in decision making forums, and barriers to participation in regional and international professional networks (21). The described constraints among women in oncology are likely to be present in other medical subspecialties. A review of female surgeons in the Arab world identified recurring themes of gender based discrimination, patient male-doctors preferences, and family role expectations, all of which can influence female physicians readiness to pursue senior roles (22).

From a health policy perspective, an important implication of the inverted gender pyramid is that nations investments in medical education may not necessarily translate into returns in senior clinical leadership capacity when a large proportion of trained women doctors are underutilized in decision making roles. In GCC countries, leadership gender diversity will contribute positively to the enhancement of problem solving, innovation, and responsiveness to patient and health workforce needs. Evidence from the private health sector similarly supports a system performance rationale for improving women’s representation in senior leadership positions (23). We believe that gender equity in career progression should be an integral part of health system performance management and key performance indicators.

Strengths of this study arises from the use of a sizeable analytic sample with standardized variables for gender, nationality, career level, and years of service. The employment of multivariable models has allowed for the estimation of the independent associations between gender and advanced career attainment. The complete case analysis for key career variables has ensured alignment with credible results. However, these findings should be interpreted in light of limitations. The cross-sectional design cannot establish causality. Career level is used as a proxy for career advancement, yet it may not reflect leadership titles, academic rank, specialty distribution and sector of employment. In addition, while GCC nationality was included as a contextual variable, the collected data did not allow analysis within country heterogeneity.

In conclusion, an inverted gender pyramid exists in career progression among GCC physicians. Women comprise the majority of medical graduates, yet men are more likely to occupy advanced career levels, and this association persists after accounting for years in medical career, experience and nationality. This pattern is consistent with increasing international evidence that gender equity in entry to medical schools does not automatically translate after graduation into equity in leadership and senior roles. These findings indicate the urgent need for health policy action that improves promotion transparency, formalizes leadership pipelines, and expands enabling policies that support equitable physicians career progression.

## Data Availability

The data analyzed in this study is subject to the following licenses/restrictions: Datasets are available on request: The raw data supporting the conclusions of this article will be made available by the authors, without undue reservation. Requests to access these datasets should be directed to salmanhz@agu.edu.bh.
